# Oxaliplatin and protracted venous infusion of 5-fluorouracil in patients with advanced or relapsed 5-fluorouracil pretreated colorectal cancer

**DOI:** 10.1054/bjoc.2001.2036

**Published:** 2001-09-01

**Authors:** I Chau, A Webb, D Cunningham, M Hill, J S Waters, A Norman, A Massey

**Affiliations:** Gastrointestinal Unit, Department of Medicine, Royal Marsden Hospital, Downs Road, Sutton, Surrey, SM2 5PT; Department of Computing and Information, Royal Marsden Hospital, Downs Road, Sutton, surrey, SM2 5PT, UK

**Keywords:** oxaliplatin, 5-fluorouracil, protracted venous infusion, colorectal carcinoma

## Abstract

The purpose of this study was to evaluate the activity and safety of oxaliplatin and protracted venous infusion of 5-fluorouracil (PVI 5-FU) in patients with advanced or relapsed 5-FU pretreated colorectal cancer. 38 patients with advanced or metastatic colorectal carcinoma with documented progression on or within 6 months following 5-FU or thymidylate synthase inhibitor containing chemotherapy were recruited between June 1997 and September 2000. Oxaliplatin (100 mg m^−2^) was given every 2 weeks and PVI 5-FU (300 mg m^−2^day^−1^) was administered. Median age of patients was 61 years. 17 patients had >2 sites of disease involvement. 10 had received 5-FU based adjuvant chemotherapy. 16 received oxaliplatin and PVI 5-FU as second-line chemotherapy for advanced disease and 22 as third or subsequent lines. Median follow up was 6.1 months. The best achieved objective tumour response rate was 29% (11 partial responses 95% confidence interval [CI] = 15–46%). 20 patients (52.6%) had stable disease. The median duration of response was 3.9 months. Even for patients who had previously received both 5-FU and irinotecan (*n*= 22), 27.3% had partial response with oxaliplatin and PVI 5-FU. 37 patients had symptoms on entry into the study. 25 patients had pain, 10 had anorexia and 28 had lethargy. 64%, 70% and 17.9% had symptomatic improvement after treatment respectively. Grade 3–4 toxicities were anaemia 10.6%, neutropenia 2.6%, thrombocytopenia 5.2%, diarrhoea 18.9%, nausea and vomiting 2.7%, infection 5.4% and lethargy 37.8%. The median survival was 9.1 months. Probability of overall survival at 6 months was 58.4% (95% CI = 38.7–73.7%). The median failure-free survival was 4 months. Oxaliplatin and PVI 5FU is an active and well tolerated regimen in patients with heavily pre-treated advanced colorectal cancer. © 2001 Cancer Research Campaign


*British Journal of Cancer* (2001) **85**(9), 1258–1264
© 2001 Cancer Research Campaign
doi: 10.1054/ bjoc.2001.2036, available online at http://www.idealibrary.com on IDEAL

http://www.bjcancer.com

### Oxaliplatin and protracted venous infusion of 5-fluorouracil in patients with advanced or relapsed 5-fluorouracil pretreated colorectal cancer

**I Chau[1], A Webb[1], D Cunningham[1], M Hill[1], JS Waters[1], A Norman[2] and A Massey[1]**

[1]Gastrointestinal Unit, Department of Medicine, Royal Marsden Hospital, Downs Road, Sutton, Surrey, SM2 5PT; [2]Department of Computing and Information, Royal Marsden Hospital, Downs Road, Sutton Surrey, SM2 5PT UK

**Summary** The purpose of this study was to evaluate the activity and safety of oxaliplatin and protracted venous infusion of 5-fluorouracil (PVI 5-FU) in patients with advanced or relapsed 5-FU pretreated colorectal cancer. 38 patients with advanced or metastatic colorectal carcinoma with documented progression on or within 6 months following 5-FU or thymidylate synthase inhibitor containing chemotherapy were recruited between June 1997 and September 2000. Oxaliplatin (100 mg $m^{-2}$) was given every 2 weeks and PVI 5-FU (300 mg $m^{-2}$ $day^{-1}$) was administered. Median age of patients was 61 years. 17 patients had >2 sites of disease involvement. 10 had received 5-FU based adjuvant chemotherapy. 16 received oxaliplatin and PVI 5-FU as second-line chemotherapy for advanced disease and 22 as third or subsequent lines. Median follow up was 6.1 months. The best achieved objective tumour response rate was 29% (11 partial responses 95% confidence interval [CI] = 15–46%). 20 patients (52.6%) had stable disease. The median duration of response was 3.9 months. Even for patients who had previously received both 5-FU and irinotecan ($n$ = 22), 27.3% had partial response with oxaliplatin and PVI 5-FU. 37 patients had symptoms on entry into the study. 25 patients had pain, 10 had anorexia and 28 had lethargy. 64%, 70% and 17.9% had symptomatic improvement after treatment respectively. Grade 3–4 toxicities were anaemia 10.6%, neutropenia 2.6%, thrombocytopenia 5.2%, diarrhoea 18.9%, nausea and vomiting 2.7%, infection 5.4% and lethargy 37.8%. The median survival was 9.1 months. Probability of overall survival at 6 months was 58.4% (95% CI = 38.7–73.7%). The median failure-free survival was 4 months. Oxaliplatin and PVI 5FU is an active and well tolerated regimen in patients with heavily pre-treated advanced colorectal cancer. © 2001 Cancer Research Campaign

**Keywords**: oxaliplatin; 5-fluorouracil; protracted venous infusion; colorectal carcinoma

Colorectal cancer (CRC) is the second most common cause of cancer death in the Western world. 5-year survival is poor ranging from 40 to 60% (Office for National Statistics, 1999). However 25% of patients present with metastatic disease with dismal outcome and ultimately 50% of patients will die of locally advanced or metastatic disease. Protracted intravenous infusion (PVI) of 5 fluorouracil (5-FU) produced a modest response rate in patients who were chemonaive (30%) (Lokich et al, 1989) or resistant to bolus 5-FU (22%) (Lokich et al, 1983). Continuous infusion of 5-FU has shown a small but statistically significant survival advantage over bolus protocol in a recent meta-analysis (The Advanced Colorectal Cancer Meta-Analysis Project, 1998) and has been the mainstay salvage treatment for bolus 5-FU resistant colorectal carcinoma patients until recently. Oxaliplatin is a novel platinum compound with promising results in first-line treatment for metastatic colorectal carcinoma in combination with fluoropyrimidines. 2 randomised trials in untreated patients with advanced colorectal carcinoma have shown superior response with combination of oxaliplatin and 5-FU/leucovorin (LV) over the same schedule of 5-FU/LV (de Gramont et al, 2000; Giacchetti et al, 2000). Both of these trials have also shown a significant progression-free survival benefit with oxaliplatin but no overall survival advantage. However, a large proportion of patients in the 5-FU/LV alone arm crossed over to oxaliplatin salvage or received irinotecan, which is a proven effective second-line treatment (Cunningham et al, 1998; Rougier et al, 1998). Since it is likely that patients have few symptoms prior to tumour progression, progression-free survival would probably be an adequate surrogate marker of the true clinical benefit derived from oxaliplatin. Neither of these trials reported on the symptoms patients experienced before, during or after chemotherapy, therefore no direct evidence of palliative effect on patients' symptoms can be obtained. Although quality of life (QoL) data were reported in one trial (de Gramont et al, 2000), no definite advantage was seen with oxaliplatin (only time to deterioration of the global health status was significantly prolonged in the oxaliplatin arm). However, no deterioration in QoL was seen despite higher incidences of toxicity associated with oxaliplatin over 5-FU/LV alone. This is likely to reflect oxaliplatin's positive impact on tumour-related symptoms.

Oxaliplatin and 5-FU have also been tested in a number of second-line studies in advanced colorectal carcinoma (Machover et al, 1996; Andre et al, 1998, 1999; Brienza et al, 1999; Maindrault-Goebel et al, 1999, 2001; Adenis et al, 2000; Martoni et al, 2001). No randomised phase III trials have yet been published in full, therefore no conclusions can be drawn from them although the preliminary data do look promising (Adenis et al, 2000).

*Received 19 January 2001*
*Revised 21 June 2001*
*Accepted 4 July 2001*

*Correspondence to:* D Cunningham

Presented at the 37th Annual Meeting of the American Society of Clinical Oncology, May 2001.

The safety and efficacy of a variety of dosing regimens with 5-FU and leucovorin and oxaliplatin have been evaluated and the combination has demonstrated marked antitumour efficacy (Levi et al, 1994, 1997, 1999; Machover et al, 1996; Andre et al, 1998, 1999; Gramont et al., 1997, 2000; Giacchetti et al, 2000). Oxaliplatin and PVI 5-FU (OXAF), however, has not been extensively explored and we have therefore investigated this combination using a 2 weekly schedule of oxaliplatin in 5-FU resistant patients.

Prior to the commencement of our study, FOLFOX 2 regimen was reported with a response rate of 46% and a median progression-free survival of 7 months in patients who progressed while on leucovorin and 5-FU therapy (de Gramont et al, 1997). This 2-weekly regimen consisted of oxaliplatin 100 mg $m^{-2}$ as a 2-hour infusion on day 1, leucovorin 500 mg $m^{-2}$ $day^{-1}$ as a 2-hour infusion followed by a 24-hour infusion of 5-FU at 1500–2000 mg $m^{-2}$ $day^{-1}$ for 2 consecutive days. Based on the encouraging results from FOLFOX 2, biweekly oxaliplatin at a dose of 100 mg $m^{-2}$ was adopted in our study.

### PATIENTS AND METHOD

We conducted a phase II open-labelled study between June 1997 and September 2000. This study was approved by the local biomedical ethics committees. A signed, written informed consent was obtained from each patient.

### Eligibility criteria

Admission criteria were: histologically proven advanced or metastatic inoperable adenocarcinoma of the colon or rectum; documented progression during or within 6 months following fluoropyrimidine or thymidylate synthase inhibitor containing chemotherapy for advanced disease or during 5-FU based adjuvant treatment; bidimensionally measurable disease on chest X-ray or computer tomography (CT scan) outside any previous irradiated zone; ECOG performance status 0–2; absence of severe neuropathy (> grade 1 according to National Cancer Institute-Common Toxicity Criteria (Macdonald et al, 1995)) and no central nervous system disease. Baseline criteria of blood analyses were as follows: white blood count $>3 \times 10^9$ $l^{-1}$, neutrophil $> 1.5 \times 10^9$ $l^{-1}$ and platelet $> 100 \times 10^9$ $l^{-1}$, bilirubin <30 µmol $l^{-1}$, creatinine <180 µmol $l^{-1}$. Before entry into the study, all patients were required to have chest X-ray, CT scan of chest, abdomen and pelvis and carcinoembryonic antigen (CEA) measurement.

### Chemotherapy regimen

Oxaliplatin (100 mg $m^{-2}$) was delivered as an intravenous infusion over 2 hours. This was repeated every 2 weeks unless there were significant toxicities (one cycle). No pre- or post-chemotherapy hydration was required for oxaliplatin administration. 5-FU (300 mg $m^{-2}$ $day^{-1}$) was administered as a continuous infusion via a central venous catheter (Hickman line). Patients who had previous toxicity on PVI 5-FU requiring early dose reduction (within 4 weeks of starting treatment) started 5-FU at that reduced dose. A maximum of 24 weeks of treatment was given.

Antiemetic regimen included dexamethasone 8 mg and granisetron 1 mg both as bolus intravenous injection prior to each oxaliplatin administration. In addition metoclopromide 20 mg 4 times per day for 3 days and dexamethasone 4 mg 3 times per day for 2 days orally were given after each oxaliplatin administration.

### Dose modification

Toxicities were assessed according to National Cancer Institute-Common Toxicity Criteria (NCI-CTC) version 1 (Macdonald et al, 1995). If thrombocytopenia or leucopenia > grade 3 developed during the study, oxaliplatin was delayed until recovery and reduced to 80 mg $m^{-2}$ on subsequent cycles of treatment. PVI 5-FU was stopped if grade 4 thrombocytopenia or neutropenia occurred. Oxaliplatin was reduced to 80 mg $m^{-2}$ on subsequent cycles of treatment if grade 2 neuropathy occurred. If ≥ grade 3 neuropathy was encountered, oxaliplatin would be discontinued.

If stomatitis, hand–foot syndrome or diarrhoea relating to 5-FU developed, 50 mg $m^{-2}$, 100 mg $m^{-2}$ and 150 mg $m^{-2}$ dose reductions were made to 5-FU if grade 2, 3 and 4 toxicities developed respectively. Chemotherapy was discontinued in the events of disease progression after a minimum of 4 weeks' treatment or intolerable toxicity.

### Study parameters

Toxicity assessment, physical examination, complete blood count, renal and liver function tests were performed before each cycle. Tumour-related symptoms were assessed with a 15-point checklist at baseline and at each hospital visit for patients who had these symptoms on entry into the study. Disappearance or attenuation of these tumour-related symptoms was recorded at each hospital visit. CEA was measured at least once every 8 weeks. A reduction in CEA concentration was considered a biological effect in patients whose CEA levels had been elevated at baseline, but was not used to evaluate response. Antitumour activity was evaluated according to World Health Organisation (WHO) criteria (Miller et al, 1981). CT scans of thorax, abdomen and pelvis were repeated every 4 cycles or earlier in cases of clinical deterioration. External review and confirmatory CT scans were not performed.

Complete response (CR) was defined as the complete disappearance of all measurable lesions, without the appearance of new lesion(s). Partial response (PR) was defined as a reduction of bidimensional lesions by ≥ 50% of the sum of the products of the largest perpendicular diameters of each measurable lesion and no progression in other lesions or the appearance of any new lesions. Stable disease (SD) was defined as a < 50% reduction of tumour volume or a less than 25% increase of the volume of one or more measurable lesions, with no new lesions. Progressive disease (PD) was defined as an increase of ≥ 25% of the size of at least one bidimensionally measurable lesion, the appearance of new lesion(s), and/or the onset of ascites or pleural effusion with cytological confirmation.

### Statistical considerations

Gehan's 2-stage design was employed for estimating the response rate. The sample size calculation was based on the double requirement of being able to stop the study early if the response rate was lower than 20% and of estimating the response rate with a standard error of < 0.1, power 95%. During the first stage of the design comprising the first 14 patients, at least 4 responses were found. We therefore proceeded to the second stage when response could then be estimated with a standard error of < 0.1, power 95%.

Failure-free survival and overall survival were estimated using the Kaplan–Meier method from the start of chemotherapy. All endpoints were updated on 1 October, 2000. Failure-free survival

© 2001 Cancer Research Campaign was calculated from the date chemotherapy was started to the date either disease progression or death was observed. Overall survival was estimated from the date of chemotherapy started to the date of death from any cause. All analyses were made on an intention-to-treat basis.

### RESULTS

From June 1997 to September 2000, 38 patients were enrolled into the study. One patient did not have objective tumour response and toxicity assessment because of rapidly progressive disease. This patient was considered to have progressed from the date of starting chemotherapy. She was included in the survival and tumour response analyses on intention to treat basis. However, she was excluded from toxicity analysis.

One patient had performance status of 3 on entry, but was included in all analyses. Median follow up of patients was 6.1 months. Their characteristics are shown in Table 1. Median age of patients was 61 years. 45% of patients had more than 2 sites of disease involvement with liver being the most commonly affected. 74% of patients had locally advanced or metastatic disease at the time of diagnosis. 35 patients had elevated CEA on entry into the study with 45% > 100 μg $l^{-1}$.

10 patients had received 5-FU-based adjuvant chemotherapy in the past. A majority of patients received oxaliplatin and PVI 5-FU as third or subsequent lines of palliative chemotherapy. 22 patients had previously received both 5-FU and irinotecan as palliative regimens (see Table 2). Out of these 22 patients, 13 had progressive disease while receiving irinotecan. 7 patients had a response duration ≤ 3 months on irinotecan. One patient was intolerant to irinotecan and one received mitomycin C in between irinotecan and oxaliplatin.

**Table 1** Patients characteristics

| | |
|---|---|
| Number of patients included | 38 |
| Median age | 61 (19–75) |
| Gender | |
| Male | 24 |
| Female | 14 |
| Primary tumour | |
| Colon | 26 |
| Rectum | 10 |
| Rectosigmoid junction | 2 |
| Sites of metastasis | |
| Liver | 27 |
| Lung | 17 |
| Others | 17 |
| Involved sites | |
| 1 | 7 |
| 2 | 14 |
| >2 | 17 |
| WHO performance status | |
| 0 | 3 |
| 1 | 22 |
| 2 | 12 |
| 3 | 1 |
| Disease status at first diagnosis | |
| Advanced disease | 28 |
| Localised disease | 10 |
| Elevated alkaline phosphatase | |
| > 1 × ULN* | 20 |
| > 3 × ULN* | 6 |
| Elevated CEA | |
| > 5 μg $l^{-1}$ | 35 |
| > 100 μg $l^{-1}$ | 17 |

*ULN = upper limit of normal range.

**Table 2** Chemotherapy history

| | |
|---|---|
| Adjuvant chemotherapy (*n* = 10) | |
| Bolus e.g. NCCTG[a] regimen | 7 |
| Infused 5-FU | 3 |
| Lines of palliative chemotherapy | |
| Second line chemotherapy[b] | 16 |
| ≥ Third line chemotherapy | 22 |
| **Previous palliative chemotherapy regimens** | |
| Infused 5-FU alone | 9 |
| 5-FU/leucovorin bolus | 5 |
| 5-FU/leucovorin + experimental tumour vaccine | 3 |
| Irinotecan | 12 |
| Irinotecan/ (modified) de Gramont 5-FU[c]/leucovorin | 6 |
| Irinotecan + raltitrexed | 3 |
| Irinotecan + UFT | 2 |

[a]NCCTG = North Central Cancer Treatment Group regimen refers to bolus 5-FU day 1–5 every 4 weeks. [b]Two patients developed metastatic disease during adjuvant 5-FU based treatment and their adjuvant therapy was considered as their first palliative line of chemotherapy. [c]de Gramont 5-FU regimen refers to bolus 5FU on day 1+2 followed by 24 hours' infusion of 5-FU on each day repeated every 14 days. Modified de Gramont 5-FU regimen refers to bolus 5FU on day 1 only followed by 48 hours' infusion of 5-FU repeated every 14 days.

### Toxicity

The incidences of the main toxic effects per patient according to NCI-CTC grades are listed in Table 3. 252 cycles were evaluated. The median number of cycles administered per patient was 6. Grade 3–4 neutropenia occurred in only 2.6% of patients without any episodes of febrile neutropenia. The only significant nonhaematological toxicities were lethargy (37.8%) and diarrhoea (18.9%). More encouragingly, no grade 3/4 neurological deficit was noted in any participant although all patients who developed a mild degree of peripheral sensory neuropathy responded well to dose reduction. Only 4 patients required dose reduction due to neuropathy after a median of 3 courses of oxaliplatin. 4 patients had Hickman line-related complications. They were all superficial Hickman-line exit-site infections that responded to a course of oral flucloxacillin. No other Hickman-line-related complications were noted.

There was a significant increase in serum creatinine with OXAF treatment (paired-samples *t*-test $P = 0.001$), but the mean of maximum serum creatinine during treatment was only 103 μmol $l^{-1}$. Altogether 8 patients developed a rise in serum creatinine above the upper limit of normal (ULN) during OXAF treatment. Only 2 out of 8 patients developed grade 1 toxicity (serum creatinine > 1.5 × ULN). Both of these episodes occurred as terminal events on the dates of death for both patients. Consequently, no dose delay or reductions due to renal dysfunction were necessary during the study. No patients were withdrawn from the study due to intolerable toxicities.

### Tumour response

The best achieved objective response rate (ORR) for all patients was 29% (95% Confidence Interval (CI) = 15–46%). 11 partial

© 2001 Cancer Research Campaign

**Table 3** Toxicity profile

| Side effects | NCI-CTC* grade (%) | | | | |
|---|---|---|---|---|---|
| | 0 | 1 | 2 | 3 | 4 |
| Diarrhoea | 29.7 | 18.9 | 32.4 | 10.8 | 8.1 |
| Stomatitis | 62.2 | 24.3 | 10.8 | 2.7 | 0 |
| Nausea and vomiting | 24.3 | 43.2 | 29.7 | 2.7 | 0 |
| Alopecia | 64.9 | 16.2 | 18.9 | 0 | 0 |
| Peripheral neuropathy | 0 | 66.7 | 33.3 | 0 | 0 |
| Palmar plantar erythema | 62.2 | 21.6 | 16.2 | 0 | 0 |
| Infection | 64.9 | 10.8 | 18.9 | 5.4 | 0 |
| Fever | 83.8 | 2.7 | 10.8 | 2.7 | 0 |
| Lethargy | 0 | 8.1 | 54.1 | 37.8 | 0 |
| Anaemia | 36.8 | 10.5 | 42.1 | 5.3 | 5.3 |
| Neutropenia | 71.1 | 18.4 | 7.9 | 2.6 | 0 |
| Thrombocytopenia | 55.3 | 21.1 | 18.4 | 2.6 | 2.6 |

*NCI-CTC = National Cancer Institute-Common Toxicity Criteria. All 38 patients assessable for haematological toxicities. 37 assessable for non-haematological toxicities.

**Table 4** Disease response

| Response | Number of patients ($n$ = 38) | % of patients |
|---|---|---|
| Complete response | 0 | 0 |
| Partial response | 11 | 28.9 |
| Stable disease | 20 | 52.6 |
| Progressive disease | 7 | 18.4 |

responses and no complete responses were noted (see Table 4). The median duration of response was 3.9 months. Even for patients who had previously received both 5-FU and irinotecan ($n$ = 22), 27.3% had partial response with OXAF.

### Symptom response

37 patients had symptoms on entry into the study. 25 patients had pain, 10 had anorexia and 28 had lethargy. 64%, 70% and 17.9% had symptomatic improvement after treatment respectively. 10 patients experienced weight loss prior to receiving OXAF and all of them had weight stabilisation or weight gain following treatment. 35 patients had elevated CEA on entry into study. There was a statistically significant decrease in CEA with treatment (paired-samples $t$-test $P$ = 0.022).

### Survival

The median failure-free survival was 4 months (see Figure 1). The median overall survival was 9.1 months. The survival probability at 6 months was 58.4% (95% CI = 38.7–73.7%), at 9 months was 39.4% (95% CI = 14.6–63.7%) and at 12 months was 0% (see Figure 2). For patients who had received both irinotecan and 5-FU ($n$ = 22), the median survival was 6 months. The survival probability at 6 months was 47.9% (95% CI = 23–69.2%).

### Dose intensity

### *Oxaliplatin*

Eight patients (21%) had dose reductions in oxaliplatin during the study. Only one patient required more than one dose reduction due to toxicity (persistent thrombocytopenia). Therefore 97% of patients had ≥ 80% of the intended dose of oxaliplatin.

### *PVI 5-FU*

31 patients (82%) started 5-FU at 300 mg $m^{-2}$ $day^{-1}$, 22 (58%) patients required dose reduction during the study. The median dose of 5-FU on finishing treatment was 225 mg $m^{-2}$ $day^{-1}$. 20 out of 31 patients starting at 300 mg $m^{-2}$ $day^{-1}$ required dose modification

### DISCUSSION

With the introduction of new drugs such as irinotecan and oxaliplatin in metastatic CRC in the last decade, oncologists now have an improved armoury against this second most common cancer killer in the Western world. However oxaliplatin has a limited single agent activity against metastatic colorectal carcinoma (Machover et al, 1996) and the challenge remains to determine the optimal dose and schedule of oxaliplatin in combination with 5-FU and other drugs such as oral fluoropyrimidines (Zeuli et al, 2000; Cunningham and James, 2001), irinotecan (Wasserman et al, 1999; Goldwasser et al, 2000; Gornet et al, 2000; Hejna et al, 2000) and other TS inhibitors (Douillard et al, 2000; Fizazi et al, 2000; Scheithauer et al, 2001). Several phase II and III studies have confirmed the activity of oxaliplatin and 5-FU with folinic acid either as constant (Levi et al, 1994; Andre et al, 1999; Maindrault-Goebel et al, 1999) or chronomodulated continuous infusion (Levi et al, 1994, 1997, 1999; Giacchetti et al, 2000). 2 studies reported on the use of oxaliplatin and protracted venous infusion of 5-FU. Martoni et al recently reported a response rate of 22% in 50 patients who were treated with a 3-weekly schedule of oxaliplatin and PVI 5-FU (Martoni et al, 2001). In this study, patients were treated at 4 dose levels employing 2 different doses of oxaliplatin and 2 doses of 5-FU. The authors suggested the optimum dose level of oxaliplatin should be 130 mg $m^{-2}$ every 3 weeks and PVI 5-FU should be 250 mg $m^{-2}$ $day^{-1}$. Only 16 patients were treated at this optimum dose, although a higher response rate (37.5%) was achieved. Interestingly, for patients who had received 2 or more lines of antitumour treatment in this study, only one responder was seen. In another study, Adenis et al reported a 36.7% response rate using a 3-weekly schedule of oxaliplatin 130 mg $m^{-2}$ and 5-FU 250 mg $m^{-2}$ $day^{-1}$ in a phase II–III study

© 2001 Cancer Research Campaign 

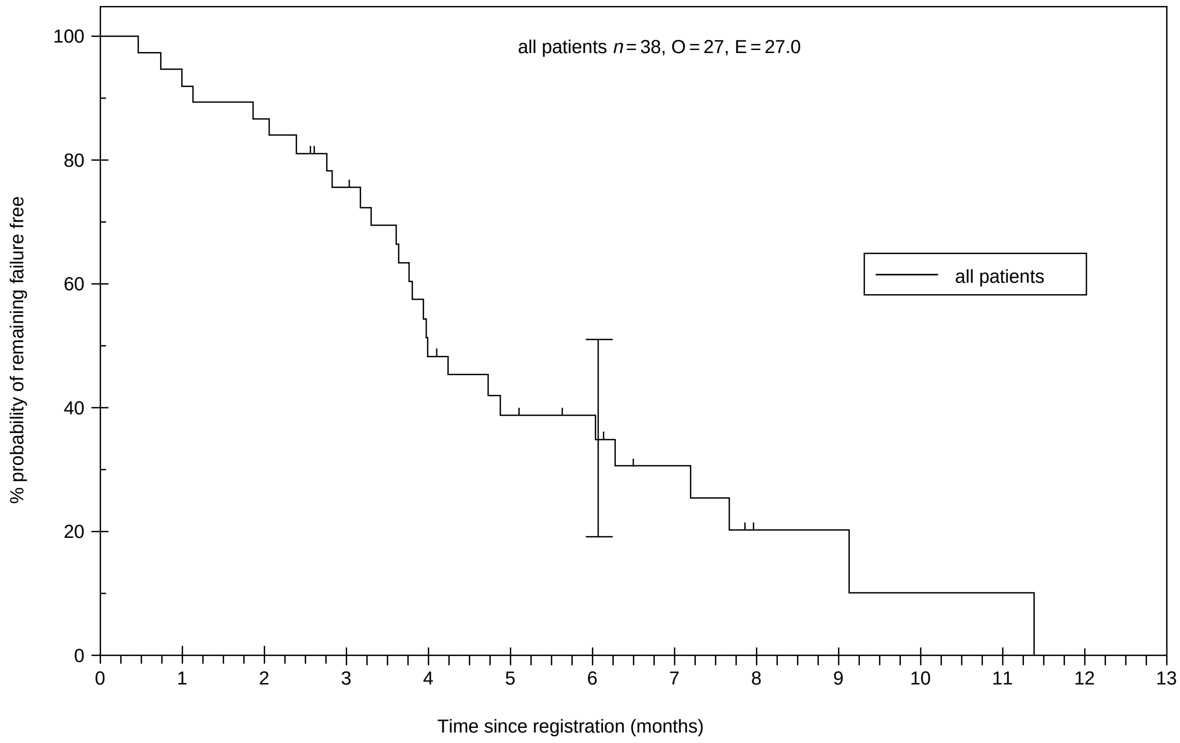

**Figure 1** The median failure-free survival was 4 months

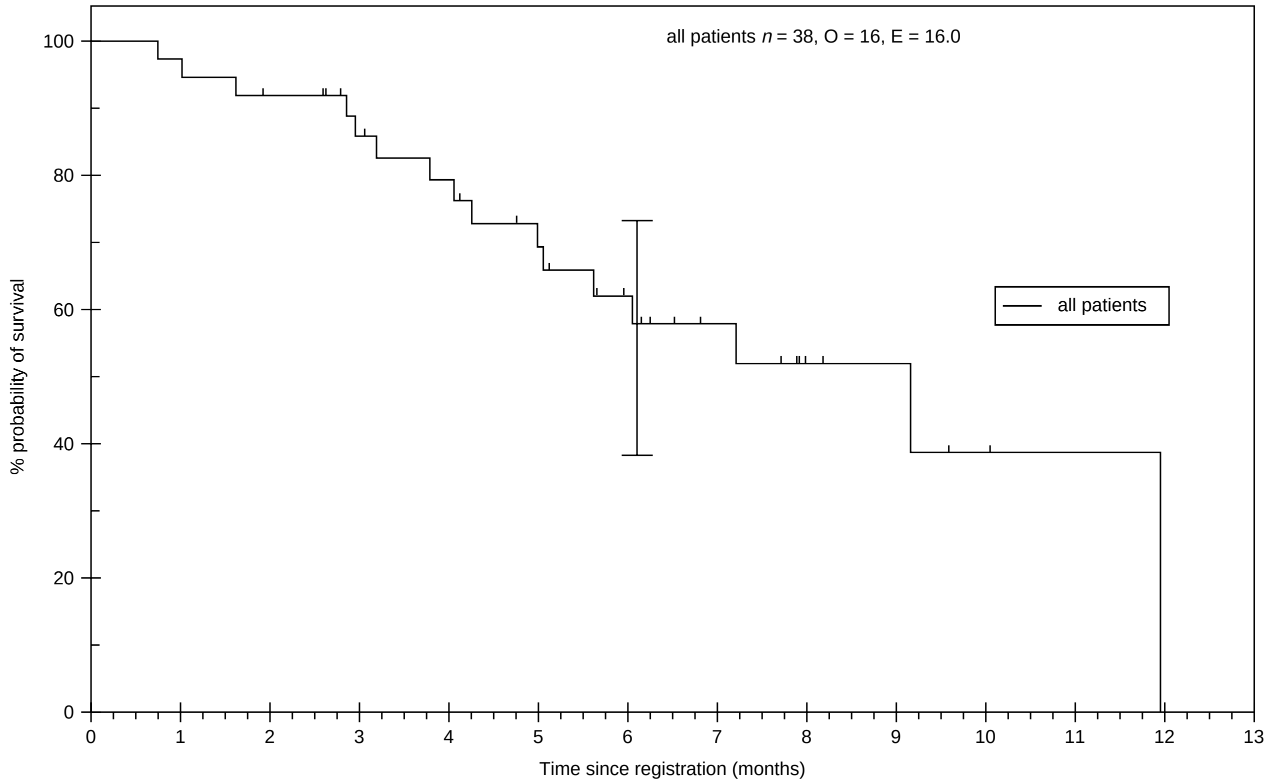

**Figure 2** The survival probability at 6 months was 58.4% (95% CI = 38.7–73.7%), at 9 months was 39.4% (95% CI = 14.6–63.7%) and at 12 months was 0%

 *© 2001 Cancer Research Campaign*

comparing this regimen with either PVI 5-FU or irinotecan (Adenis et al, 2000). Final results of the second study are awaited.

Our study has confirmed that OXAF was an active and safe regimen. In particular, our cohort of patients had a heavy tumour burden and had been heavily pre-treated before. 82% of patients had 2 or more sites of metastasis on entry into study. All but 3 had elevated CEA. Moreover, over half of patients received OXAF as third or subsequent lines of palliative chemotherapy with 22 patients received prior fluoropyrimidines and irinotecan chemotherapy either in combination or in sequential fashion.

Despite these adverse factors, a response rate of 29% was still achieved which compared favourably with other second-line studies in advanced CRC using oxaliplatin, 5-FU and leucovorin. Furthermore, another 53% achieved stable disease. Disease stabilisation is clinically meaningful as quality of life benefits have been shown in those in whom the disease is stabilised (Glimelius et al, 1994; Allen et al, 1998; Van Cutsem et al, 1999). Other studies have shown that stabilisation of progressive CRC was associated with both prolonged survival and subjective improvement (Allen et al, 1998). In our cohort of patients, over 80% of patients had control of tumour growth from receiving OXAF and might therefore have derived clinical benefit. This was further evidenced by the high proportion of patients who obtained symptomatic improvement following treatment in the study. Moreover, the survival rate in our study is comparable to other second line studies using either oxaliplatin or irinotecan based chemotherapy (Cunningham et al, 1998; Rougier et al, 1998; Andre et al, 1999; Brienza et al, 1999; Maindrault-Goebel et al, 1999].

The encouraging results from our study may be explained by the fact that nearly 80% of patients had full intended dose of oxaliplatin. Another encouraging observation was the relatively high response rate after both 5-FU and irinotecan failures. Few published studies have reported activity of oxaliplatin after irinotecan failure and the relatively high response rate demonstrated non-cross resistance of these 2 useful drugs in advanced CRC. As the patient number in this subgroup of our study was small ($n$ = 22), the confidence interval of survival is understandably large. Therefore the true survival effect after irinotecan failure cannot be determined in our study.

Treatment-related toxicity during this study was mild and infrequent. No patients were withdrawn from the study due to intolerable toxicities. No functional neurological deficit was noted which was commonly the reason of patient withdrawal or dose reduction in other studies using oxaliplatin (Andre et al, 1999; Levi et al, 1999; Maindrault-Goebel et al, 1999; Giacchetti et al, 2000). Of the 8 patients that required dose reduction in oxaliplatin during the study, 4 did so because of persistent grade 2 peripheral neuropathy in-between courses. However all improved after dose adjustment and no further dose reductions were required. Severe dose-limiting peripheral sensory neuropathy (grade 3/4) normally occurs in 10% to 15% of patients after a total cumulative dose of 780 to 850 mg $m^{-2}$ of oxaliplatin (Armand et al, 2000). As the median number of oxaliplatin courses in our study was 6 giving a cumulative dose of 600 mg $m^{-2}$, this may have explained the low incidence of peripheral sensory neuropathy. There was a statistically significant deterioration of renal function demonstrated by increase in serum creatinine in participants during the study, but the mean maximum serum creatinine after treatment was only 103 μmol $l^{-1}$. Since we have not directly measured glomerular filtration rate (GFR) during this study, the renal dysfunction demonstrated did not appear to be clinically relevant. Pharmacokinetic studies in 5-FU and oxaliplatin indicated no significant interactions occurred between these 2 drugs (Misset and Allain, 1995; Papamichael et al, 1998). Moreover glomerular filtration is the principal mechanism of platinum clearance after oxaliplatin administration and the renal clearance of ultrafilterable platinum has been shown to be significantly correlated with GFR (Graham et al, 1999, 2000). Glomerular filtration rate measurement would therefore be a more accurate assessment of renal toxicity from oxaliplatin administration.

Incidences of grade 3/4 haematological toxicity, especially neutropenia, during this study were infrequent compared to other studies (Andre et al, 1999; Levi et al, 1999; Maindrault-Goebel et al, 1999; de Gramont et al, 2000; Giacchetti et al, 2000) considering many patients have been heavily pre-treated. This is presumably due to the nonmyelosuppressive nature of PVI 5-FU. The only significant 5-FU-related toxicity was diarrhoea. Nearly two thirds of participants required some dose reduction of 5-FU during our study with a starting dose of 300 mg $m^{-2}$ $day^{-1}$. Therefore a starting dose of 250 mg $m^{-2}$ $day^{-1}$ could be considered in this biweekly oxaliplatin regimen especially in patients who have previously developed early toxicity to 5-FU.

In conclusion oxaliplatin and PVI 5-FU is an active and well-tolerated regimen in patients with heavily pre-treated advanced colorectal cancer. Furthermore, OXAF remains a viable and attractive option after irinotecan failure. This regimen should be further evaluated in both first-and second-line settings.

### REFERENCES

Adenis A, Douillard JY, Lacroix H, Dufour P, Tubiana N, Berger C, Bugat R, Mousseau M, Block S and Dupont-Andre G (2000) Randomized phase II-III trial of oxaliplatin (OXA) with 5-fluorouracil continuous infusion versus a control arm with either 5FU or irinotecan in previously treated metastatic colorectal cancer patients. *Proc Am Soc Clin Oncol* **19**: 279a

Allen M, Cunningham D and Schmitt C (1998) The importance of stabilization as an endpoint in the treatment of metastatic colorectal carcinoma: recent quality of life studies. *Anticancer Drugs* **9**: 783–790

Andre T, Louvet C, Raymond E, Tournigand C and de Gramont A (1998) Bimonthly high-dose leucovorin, 5-fluorouracil infusion and oxaliplatin (FOLFOX3) for metastatic colorectal cancer resistant to the same leucovorin and 5-fluorouracil regimen. *Ann Oncol* **9**: 1251–1253

Andre T, Bensmaine MA, Louvet C, Francois E, Lucas V, Desseigne F, Beerblock K, Bouche O, Carola E, Merrouche Y, Morvan F, Dupont-Andre G and de Gramont A (1999) Multicenter phase II study of bimonthly high-dose leucovorin, fluorouracil infusion, and oxaliplatin for metastatic colorectal cancer resistant to the same leucovorin and fluorouracil regimen. *J Clin Oncol* **17**: 3560–3568

Armand JP, Boige V, Raymond E, Fizazi K, Faivre S and Ducreux M (2000) Oxaliplatin in colorectal cancer: an overview. *Semin Oncol* **27**: 96–104

Brienza S, Bensmaine MA, Soulie P, Louvet C, Gamelin E, Francois E, Ducreux M, Marty M, Andre T, de Braud F, Bleiberg H, Segal V, Itzhaki M and Cvitkovic E (1999) Oxaliplatin added to 5-fluorouracil-based therapy (5-FU+/– FA) in the treatment of 5-FU-pretreated patients with advanced colorectal carcinoma (ACRC): results from the European compassionate-use program. *Ann Oncol* **10**: 1311–1316

Cunningham D and James RD (2001) Integrating the oral fluoropyrimidines into the management of advanced colorectal cancer. *European Journal of Cancer* **37**: 826–834

Cunningham D, Pyrhonen S, James RD, Punt CJA, Hickish TF, Heikkila R, Johannesen T, Starkhammar H, Topham CA, Awad L, Jacques C and Herait P (1998) Randomised trial of irinotecan plus supportive care versus supportive care alone after fluorouracil failure for patients with metastatic colorectal cancer. *Lancet* **352**: 1413–1418

de Gramont A, Vignoud J, Tournigand C, Louvet C, Andre T, Varette C, Raymond E, Moreau S, Le Bail N and Krulik M (1997) Oxaliplatin with high-dose leucovorin and 5-fluorouracil 48-hour continuous infusion in pretreated metastatic colorectal cancer. *Eur J Cancer* **33**: 214–219

*© 2001 Cancer Research Campaign*

de Gramont A, Figer A, Seymour M, Homerin M, Hmissi A, Cassidy J, Boni C, Cortes-Funes H, Cervantes A, Freyer G, Papamichael D, Le Bail N, Louvet C, Hendler D, de Braud F, Wilson C, Morvan F and Bonetti A (2000) Leucovorin and fluorouracil with or without oxaliplatin as first-line treatment in advanced colorectal cancer. *J Clin Oncol* **18**: 2938–2947

Douillard JY, Michel P, Gamelin E, Conroy T, Francois E, Raoul JL, Becouarn Y, Cvitkovic B, Nasca S, Ychou M, Fandi A and Seitz JF (2000) Raltitrexed ('Tomudex') plus oxaliplatin: an active combination for first line chemotherapy in patients with metastatic colorectal cancer. *Proc Am Soc Clin Oncol* **19**: 250a

Fizazi K, Ducreux M, Ruffie P, Bonnay M, Daniel C, Soria JC, Hill C, Fandi A, Poterre M, Smith M and Armand JP (2000) Phase I, dose-finding, and pharmacokinetic study of raltitrexed combined with oxaliplatin in patients with advanced cancer. *J Clin Oncol* **18**: 2293–2300

Giacchetti S, Perpoint B, Zidani R, Le Bail N, Faggiuolo R, Focan C, Chollet P, Llory JF, Letourneau Y, Coudert B, Bertheaut-Cvitkovic F, Larregain-Fournier D, Le Rol A, Walter S, Adam R, Misset JL and Levi F (2000) Phase III multicenter randomized trial of oxaliplatin added to chronomodulated fluorouracil-leucovorin as first-line treatment of metastatic colorectal cancer. *J Clin Oncol* **18**: 136–147

Glimelius B, Hoffman K, Graf W, Pahlman L and Sjoden PO (1994) Quality of life during chemotherapy in patients with symptomatic advanced colorectal cancer. The Nordic Gastrointestinal Tumor Adjuvant Therapy Group. *Cancer* **73**: 556–562

Goldwasser F, Gross-Goupil M, Tigaud JM, Di Palma M, Marceau-Suissa J, Wasserman E, Yovine A, Misset JL and Cvitkovic E (2000) Dose escalation of CPT-11 in combination with oxaliplatin using an every two weeks schedule: A phase I study in advanced gastrointestinal cancer patients. *Annals of Oncology* **11**: 1463–1470

Gornet JM, Azoulay D, Levi F, Yovine A, Misset JL and Goldwasser F (2000) Dramatic tumor response of bulky liver metastases following treatment with CPT-11 and a chronomodulated 4-day infusion of 5-fluorouracil, folinic acid and oxaliplatin every 2 weeks in a colorectal cancer patient. *Anticancer Drugs* **11**: 263–268

Graham MA, Lockwood GF, Cunningham D and Gamelin E (1999) Pharmacokinetics of oxaliplatin in special patient populations. *Proc Am Soc Clin Oncol* **18**: 189.

Graham MA, Lockwood GF, Greenslade D, Brienza S, Bayssas M and Gamelin E (2000) Clinical pharmacokinetics of oxaliplatin: a critical review. *Clin Cancer Res* **6**: 1205–1218

Hejna M, Kostler WJ, Raderer M, Tomek S, Brodowicz T, Scheithauer W, Wiltschke C and Zielinski CC (2000) Phase II study of second-line oxaliplatin, irinotecan and mitomycin C in patients with advanced or metastatic colorectal cancer. *Anti-Cancer Drugs* **11**: 629–634

Levi F, Zidani R and Misset JL (1997) Randomised multicentre trial of chronotherapy with oxaliplatin, fluorouracil, and folinic acid in metastatic colorectal cancer. International Organization for Cancer Chronotherapy. *Lancet* **350**: 681–686

Levi F, Zidani R, Brienza S, Dogliotti L, Perpoint B, Rotarski M, Letourneau Y, Llory JF, Chollet P, Le Rol A and Focan C (1999) A multicenter evaluation of intensified, ambulatory, chronomodulated chemotherapy with oxaliplatin, 5-fluorouracil, and leucovorin as initial treatment of patients with metastatic colorectal carcinoma. International Organization for Cancer Chronotherapy. *Cancer* **85**: 2532–2540

Levi FA, Zidani R, Vannetzel JM, Perpoint B, Focan C, Faggiuolo R, Chollet P, Garufi C, Itzhaki M, Dogliotti L et al (1994) Chronomodulated versus fixed-infusion-rate delivery of ambulatory chemotherapy with oxaliplatin, fluorouracil, and folinic acid (leucovorin) in patients with colorectal cancer metastases: a randomized multi-institutional trial. *J Natl Cancer Inst* **86**: 1608–1617

Lokich J, Fine N, Perri J and Bothe A, Jr. (1983) Protracted ambulatory venous infusion of 5-fluorouracil. *Am J Clin Oncol* **6**: 103–107

Lokich JJ, Ahlgren JD, Gullo JJ, Philips JA and Fryer JG (1989) A prospective randomized comparison of continuous infusion fluorouracil with a conventional bolus schedule in metastatic colorectal carcinoma: a Mid-Atlantic Oncology Program Study. *J Clin Oncol* **7**: 425–432

Macdonald JS, Haller D and Mayer RJ (1995) Grading of Toxicity. In *Manual of Oncologic Therapeutics*, pp 519–523. Lippincott: Philadelphia

Machover D, Diaz-Rubio E, de Gramont A, Schilf A, Gastiaburu JJ, Brienza S, Itzhaki M, Metzger G, D ND, Vignoud J, Abad A, Francois E, Gamelin E, Marty M, Sastre J, Seitz JF and Ychou M (1996) Two consecutive phase II studies of oxaliplatin (L-OHP) for treatment of patients with advanced colorectal carcinoma who were resistant to previous treatment with fluoropyrimidines. *Ann Oncol* **7**: 95–98

Maindrault-Goebel F, Louvet C, Andre T, Carola E, Lotz JP, Molitor JL, Garcia ML, Gilles-Amar V, Izrael V, Krulik M and de Gramont A (1999) Oxaliplatin added to the simplified bimonthly leucovorin and 5-fluorouracil regimen as second-line therapy for metastatic colorectal cancer (FOLFOX6). GERCOR. *Eur J Cancer* **35**: 1338–1342

Maindrault-Goebel F, de Gramont A, Louvet C, Andre T, Carola E, Mabro M, Artru P, Gilles V, Lotz JP, Izrael V and Krulik M (2001) High-dose intensity oxaliplatin added to the simplified bimonthly leucovorin and 5-fluorouracil regimen as second-line therapy for metastatic colorectal cancer (FOLFOX 7). *Eur J Cancer* **37**: 1000–1005

Martoni A, Mini E, Pinto C, Nobili S, Gentile AL, Dentico P, Angelelli B, Scicolone S, Piana E and Mazzei T (2001) Oxaliplatin and protracted continuous 5-fluorouracil infusion in patients with pretreated advanced colorectal carcinoma. *Annals of Oncology* **12**: 519–524

Miller AB, Hoogstraten B, Staquet M and Winkler A (1981) Reporting results of cancer treatment. *Cancer* **47**: 207–214

Misset JL and Allain (1995) Pharmacokinetics, urinary, and fecal excretion of oxaliplatin in cancer patients. TDR 3500. Debiopharm/Sanofi

Office for National Statistics. Cancer Survival Trends in England & Wales 1971–1995. 1999. Office for National Statistics

Papamichael D, Joel S, Seymour M, Richards F, Bowerbank M, Slevin ML and Graham M (1998) Pharmacokinetic interaction between 5-fluorouracil and oxaliplatin. INT3681. Sanofi Research

Rougier P, Van Cutsem E, Bajetta E, Niederle N, Possinger K, Labianca R, Navarro M, Morant R, Bleiberg H, Wils J, Awad L, Herait P and Jacques C (1998) Randomised trial of irinotecan versus fluorouracil by continuous infusion after fluorouracil failure in patients with metastatic colorectal cancer. *Lancet* **352**: 1407–1412

Scheithauer W, Kornek GV, Ulrich-Pur H, Penz M, Raderer M, Salek T, Haider K, Kwasny W and Depisch D (2001) Oxaliplatin plus raltitrexed in patients with advanced colorectal carcinoma – Results of a phase I-II trial. *Cancer* **91**: 1264–1271

The Advanced Colorectal Cancer Meta-Analysis Project (1998) Efficacy of intravenous continuous infusion of fluorouracil compared with bolus administration in advanced colorectal cancer. Meta-analysis Group In Cancer. *J Clin Oncol* **16**: 301–308

Van Cutsem E, Cunningham D, Ten Bokkel Huinink WW, Punt CJ, Alexopoulos CG, Dirix L, Symann M, Blijham GH, Cholet P, Fillet G, Van Groeningen C, Vannetzel JM, Levi F, Panagos G, Unger C, Wils J, Cote C, Blanc C, Herait P and Bleiberg H (1999) Clinical activity and benefit of irinotecan (CPT-11) in patients with colorectal cancer truly resistant to 5-fluorouracil (5-FU). *Eur J Cancer* **35**: 54–59

Wasserman E, Cuvier C, Lokiec F, Goldwasser F, Kalla S, Mery-Mignard D, Ouldkaci M, Besmaine A, Dupont-Andre G, Mahjoubi M, Marty M, Misset JL and Cvitkovic E (1999) Combination of oxaliplatin plus irinotecan in patients with gastrointestinal tumors: results of two independent phase I studies with pharmacokinetics. *J Clin Oncol* **17**: 1751–1759

Zeuli M, Di Costanzo F, Sdrobolini A, Paoloni FP, Carpi A, Moscetti L, Cherubini R and Cognetti F (2000) A dose-finding study of capecitabine in combination with oxaliplatin (L-OHP) in advanced colorectal cancer (CRC). *Annals of Oncology* **11**: H61

 *© 2001 Cancer Research Campaign*

## References

[PDF_00581] Allen M., Cunningham D., Schmitt C. (1998). The importance of stabilization as an endpoint in the treatment of metastatic colorectal carcinoma: recent quality of life studies.. Anticancer Drugs.

[PDF_00588] André T., Bensmaine M. A., Louvet C., François E., Lucas V., Desseigne F., Beerblock K., Bouché O., Carola E., Merrouche Y. (1999). Multicenter phase II study of bimonthly high-dose leucovorin, fluorouracil infusion, and oxaliplatin for metastatic colorectal cancer resistant to the same leucovorin and fluorouracil regimen.. J Clin Oncol.

[PDF_00584] André T., Louvet C., Raymond E., Tournigand C., de Gramont A. (1998). Bimonthly high-dose leucovorin, 5-fluorouracil infusion and oxaliplatin (FOLFOX3) for metastatic colorectal cancer resistant to the same leucovorin and 5-fluorouracil regimen.. Ann Oncol.

[PDF_00594] Armand J. P., Boige V., Raymond E., Fizazi K., Faivre S., Ducreux M. (2000). Oxaliplatin in colorectal cancer: an overview.. Semin Oncol.

[PDF_00596] Brienza S., Bensmaïne M. A., Soulié P., Louvet C., Gamelin E., François E., Ducreux M., Marty M., André T., de Braud F. (1999). Oxaliplatin added to 5-fluorouracil-based therapy (5-FU +/- FA) in the treatment of 5-FU-pretreated patients with advanced colorectal carcinoma (ACRC): results from the European compassionate-use program.. Ann Oncol.

[PDF_00602] Cunningham D., James R. D. (2001). Integrating the oral fluoropyrimidines into the management of advanced colorectal cancer.. Eur J Cancer.

[PDF_00605] Cunningham D., Pyrhönen S., James R. D., Punt C. J., Hickish T. F., Heikkila R., Johannesen T. B., Starkhammar H., Topham C. A., Awad L. (1998). Randomised trial of irinotecan plus supportive care versus supportive care alone after fluorouracil failure for patients with metastatic colorectal cancer.. Lancet.

[PDF_00625] Fizazi K., Ducreux M., Ruffié P., Bonnay M., Daniel C., Soria J. C., Hill C., Fandi A., Poterre M., Smith M. (2000). Phase I, dose-finding, and pharmacokinetic study of raltitrexed combined with oxaliplatin in patients with advanced cancer.. J Clin Oncol.

[PDF_00629] Giacchetti S., Perpoint B., Zidani R., Le Bail N., Faggiuolo R., Focan C., Chollet P., Llory J. F., Letourneau Y., Coudert B. (2000). Phase III multicenter randomized trial of oxaliplatin added to chronomodulated fluorouracil-leucovorin as first-line treatment of metastatic colorectal cancer.. J Clin Oncol.

[PDF_00635] Glimelius B., Hoffman K., Graf W., Påhlman L., Sjödén P. O. (1994). Quality of life during chemotherapy in patients with symptomatic advanced colorectal cancer. The Nordic Gastrointestinal Tumor Adjuvant Therapy Group.. Cancer.

[PDF_00639] Goldwasser F., Gross-Goupil M., Tigaud J. M., Di Palma M., Marceau-Suissa J., Wasserman E., Yovine A., Misset J. L., Cvitkovic E. (2000). Dose escalation of CPT-11 in combination with oxaliplatin using an every two weeks schedule: a phase I study in advanced gastrointestinal cancer patients.. Ann Oncol.

[PDF_00644] Gornet J. M., Azoulay D., Lévi F., Yovine A., Misset J. L., Goldwasser F. (2000). Dramatic tumor response of bulky liver metastases following treatment with CPT-11 and a chronomodulated 4-day infusion of 5-fluorouracil, folinic acid and oxaliplatin every 2 weeks in a colorectal cancer patient.. Anticancer Drugs.

[PDF_00652] Graham M. A., Lockwood G. F., Greenslade D., Brienza S., Bayssas M., Gamelin E. (2000). Clinical pharmacokinetics of oxaliplatin: a critical review.. Clin Cancer Res.

[PDF_00655] Hejna M., Köstler W. J., Raderer M., Tomek S., Brodowicz T., Scheithauer W., Wiltschke C., Zielinski C. C. (2000). Phase II study of second-line oxaliplatin, irinotecan and mitomycin C in patients with advanced or metastatic colorectal cancer.. Anticancer Drugs.

[PDF_00675] Lokich J. J., Ahlgren J. D., Gullo J. J., Philips J. A., Fryer J. G. (1989). A prospective randomized comparison of continuous infusion fluorouracil with a conventional bolus schedule in metastatic colorectal carcinoma: a Mid-Atlantic Oncology Program Study.. J Clin Oncol.

[PDF_00673] Lokich J., Fine N., Perri J., Bothe A. (1983). Protracted ambulatory venous infusion of 5-fluorouracil.. Am J Clin Oncol.

[PDF_00668] Lévi F. A., Zidani R., Vannetzel J. M., Perpoint B., Focan C., Faggiuolo R., Chollet P., Garufi C., Itzhaki M., Dogliotti L. (1994). Chronomodulated versus fixed-infusion-rate delivery of ambulatory chemotherapy with oxaliplatin, fluorouracil, and folinic acid (leucovorin) in patients with colorectal cancer metastases: a randomized multi-institutional trial.. J Natl Cancer Inst.

[PDF_00662] Lévi F., Zidani R., Brienza S., Dogliotti L., Perpoint B., Rotarski M., Letourneau Y., Llory J. F., Chollet P., Le Rol A. (1999). A multicenter evaluation of intensified, ambulatory, chronomodulated chemotherapy with oxaliplatin, 5-fluorouracil, and leucovorin as initial treatment of patients with metastatic colorectal carcinoma. International Organization for Cancer Chronotherapy.. Cancer.

[PDF_00659] Lévi F., Zidani R., Misset J. L. (1997). Randomised multicentre trial of chronotherapy with oxaliplatin, fluorouracil, and folinic acid in metastatic colorectal cancer. International Organization for Cancer Chronotherapy.. Lancet.

[PDF_00683] Machover D., Diaz-Rubio E., de Gramont A., Schilf A., Gastiaburu J. J., Brienza S., Itzhaki M., Metzger G., N'Daw D., Vignoud J. (1996). Two consecutive phase II studies of oxaliplatin (L-OHP) for treatment of patients with advanced colorectal carcinoma who were resistant to previous treatment with fluoropyrimidines.. Ann Oncol.

[PDF_00687] Maindrault-Goebel F., Louvet C., André T., Carola E., Lotz J. P., Molitor J. L., Garcia M. L., Gilles-Amar V., Izrael V., Krulik M. (1999). Oxaliplatin added to the simplified bimonthly leucovorin and 5-fluorouracil regimen as second-line therapy for metastatic colorectal cancer (FOLFOX6). GERCOR.. Eur J Cancer.

[PDF_00692] Maindrault-Goebel F., de Gramont A., Louvet C., André T., Carola E., Mabro M., Artru P., Gilles V., Lotz J. P., Izrael V. (2001). High-dose intensity oxaliplatin added to the simplified bimonthly leucovorin and 5-fluorouracil regimen as second-line therapy for metastatic colorectal cancer (FOLFOX 7).. Eur J Cancer.

[PDF_00697] Martoni A., Mini E., Pinto C., Nobili S., Gentile A. L., Dentico P., Angelelli B., Scicolone S., Piana E., Mazzei T. (2001). Oxaliplatin and protracted continuous 5-fluorouracil infusion in patients with pretreated advanced colorectal carcinoma.. Ann Oncol.

[PDF_00701] Miller A. B., Hoogstraten B., Staquet M., Winkler A. (1981). Reporting results of cancer treatment.. Cancer.

[PDF_00710] Rougier P., Van Cutsem E., Bajetta E., Niederle N., Possinger K., Labianca R., Navarro M., Morant R., Bleiberg H., Wils J. (1998). Randomised trial of irinotecan versus fluorouracil by continuous infusion after fluorouracil failure in patients with metastatic colorectal cancer.. Lancet.

[PDF_00715] Scheithauer W., Kornek G. V., Ulrich-Pur H., Penz M., Raderer M., Salek T., Haider K., Kwasny W., Depisch D. (2001). Oxaliplatin plus raltitrexed in patients with advanced colorectal carcinoma: results of a Phase I-II trial.. Cancer.

[PDF_00725] Van Cutsem E., Cunningham D., Ten Bokkel Huinink W. W., Punt C. J., Alexopoulos C. G., Dirix L., Symann M., Blijham G. H., Cholet P., Fillet G. (1999). Clinical activity and benefit of irinotecan (CPT-11) in patients with colorectal cancer truly resistant to 5-fluorouracil (5-FU).. Eur J Cancer.

[PDF_00729] Wasserman E., Cuvier C., Lokiec F., Goldwasser F., Kalla S., Méry-Mignard D., Ouldkaci M., Besmaine A., Dupont-André G., Mahjoubi M. (1999). Combination of oxaliplatin plus irinotecan in patients with gastrointestinal tumors: results of two independent phase I studies with pharmacokinetics.. J Clin Oncol.

[PDF_00616] de Gramont A., Figer A., Seymour M., Homerin M., Hmissi A., Cassidy J., Boni C., Cortes-Funes H., Cervantes A., Freyer G. (2000). Leucovorin and fluorouracil with or without oxaliplatin as first-line treatment in advanced colorectal cancer.. J Clin Oncol.

[PDF_00610] de Gramont A., Vignoud J., Tournigand C., Louvet C., André T., Varette C., Raymond E., Moreau S., Le Bail N., Krulik M. (1997). Oxaliplatin with high-dose leucovorin and 5-fluorouracil 48-hour continuous infusion in pretreated metastatic colorectal cancer.. Eur J Cancer.

